# Comparison of accuracy of different dental age estimation methods in Finnish and Turkish populations

**DOI:** 10.2340/aos.v83.42434

**Published:** 2024-11-22

**Authors:** Aysima Darıcı, Merih Seval Ölmez, Hamdi Cem Güngör, Päivi Rajavaara, Annina Sipola, Vuokko Anttonen, Jari Päkkilä

**Affiliations:** a Department of Pediatric Dentistry, Faculty of Dentistry, Hacettepe University, Ankara, Turkey; b Division of Pediatric Dentistry, Department of Developmental Sciences, School of Dentistry, Marquette University, Milwaukee, United States; c Research Unit of Population Health, University of Oulu, Oulu, Finland; d Medical Research Center, University of Oulu, Oulu, Finland; e The Wellbeing Services County of North Ostrobothnia, Pohde, Finland; f Department of Diagnostic Radiology, University of Oulu, Oulo, Finland; g Professor Emerita, University of Oulu, Oulo, Finland; h Research Unit of Mathematical Sciences, University of Oulu, Oulu, Finland

**Keywords:** Dental age, age estimation, Cameriere’s method, Finnish children, Forensic dentistry

## Abstract

**Objective:**

The aims of this study were to assess the accuracy of Cameriere’s and Demirjian’s methods in Finnish children, and compare the findings with those of the Turkish children according to dental age.

**Material and methods:**

Dental panoramic tomography (DPT) of children (482 Finnish, 423 Turkish) aged between 5 and 15 years were evaluated. Comparison of mean difference between estimated and chronological age was evaluated. The difference between two means was analysed using paired *t*-test at 95% confidence interval (CI). Pearson correlation coefficients were used to estimate the correlation between chronological and estimated ages.

**Results:**

Demirjian’s method resulted in overestimation in all age groups except for 8-year-old girls. Dental age, however, was found to be underestimated with Cameriere’s method in all age groups but 6-year-old girls and boys. In Northern Finnish children, Demirjian’s method was more suitable for boys while Cameriere’s method led to better estimation in girls. When comparing Finnish and Turkish children, differences between dental ages and chronological ages differed significantly in 10-year-old boys and 8-year-old girls with both methods.

**Conclusion:**

Dental age of Turkish children seems higher than that of Finnish children. There is a significant difference between chronological and dental ages in both populations assessed by both methods.

## Introduction

Estimation of chronological age using morphological and radiological analysis of teeth has become essential in paediatric dentistry, orthodontics, forensic dentistry, human anthropology, and bioarcheology. Dental maturation is a complex sequence of events from initial mineralisation of a tooth, crown formation, root growth, eruption of the tooth, and root maturation [1, 2]. Maturation status of entire dentition can be evaluated for age estimation [1, 3].

Ethnicity as well as environmental factors influence growth and body composition in children and adolescents. Therefore, there is a demand for national growth references [4–6]. This is also true for dental age estimation. There are many recent studies evaluating children’s and adolescents’ dental age comparing chronological age in different populations by using different evaluation methods [7–10].

In forensic medicine, identification by dentition is essential after natural disasters such as the tsunami in 2004. Another common phenomenon is immigration. Consequently, cases occur when an individual’s age is disputed because of lost or non-existent documents. For legal purposes, accurate age determination is required. In addition to criminal cases, this is necessary for asylum seekers, refugees, and immigrants [11]. Turkish migration to Finland is increasing each year, being about 7,000 at present. It is reported that Turkish asylum seekers are the second largest group in Finland [12]. Therefore, dental age assessments of the two populations can be considered of importance.

The radiographic examination of the developmental stages of human dentition is one of the most commonly employed methods for age appraisals. Such methods are not destructive in nature and enable age estimates for both living and deceased individuals. In the last few decades, several classification systems have been proposed to grade or score the dental development phases seen on dental radiographs [13]. Several organisations such as the Study Group on Forensic Age Diagnostics and the American Society of Forensic Odontology (ASFO) recommend dental age estimation using radiographs for evaluating chronological age to deal with cases involving refugees and cheating in age-graded sports competitions [3, 13].

Finnish public health care system allows practice-based studies on patient records specifically for children and adolescents. This is because they are entitled to free dental care up to the age of 18 years, and all are patients in primary dental health care [14]. Dental care covers free orthodontics treatment for cases with considerable orthodontic problems [15]. Presence of orthodontic problems are evaluated for all children and dental panoramic tomography (DPTs) are usually taken for diagnosis and treatment planning [16]. In Türkiye, children who have government insurance receive dental care in dental faculties or hospitals. Patient records and radiographs are kept in the hospital archives.

In Finland, forensic age estimation is established on dental development [17]; therefore, the need for studies on dental age estimation is certain. Demirjian’s method, which was previously tested in the Southern Finnish population[18], has a wide acceptance in the world [19]. Demirjian’s stages which cover seven left lower and upper permanent teeth and include third molars were assessed with the method proposed by The Dental Age Research London Information Group (DARLInG) team [20]. The method was recently tested among Turkish children [21].

Cameriere’s method has also been used in studies in European countries [22–25] and in other populations [26–28]. The accuracy of Cameriere’s method was found to be acceptable in estimating Turkish children’s chronological age [29]. However, there is a knowledge gap in literature as regards the use of Cameriere’s method in Finnish population.

The present study aimed to evaluate the accuracy of Demirjian’s and Cameriere’s methods in estimating chronological age of Northern Finnish children. A second aim of this practice-based study was to compare the chronological and dental ages of children from Oulu, Finland and Ankara, Türkiye by using Demirjian’s and Cameriere’s methods.

## Materials and methods

City of Oulu, Finland, Health Services granted consent for this study on 16.09.2019 (§ 30/2019). The study protocol was also approved by the Non-Interventional Clinical Research Ethics Board of Hacettepe University, Türkiye (No: GO 19/738). This non-interventional study was based on children’s DPTs and the information about their chronological age and sex. All participants were given an ID and analyses were conducted without personal identification.

### Finnish study sample

The sample was chosen from the DPTs of children born in Finland between 2004 and 2014. DPTs of (*n* = 482) 5–15-year-old-children were randomly selected from the archives of the City of Oulu, Oral Health Section. Distribution of the participants according to sex in both populations is shown in Table 1. DPTs of those with poor image quality, any dental pathology, absence of seven left or right mandibular teeth, presenting orthodontic braces, and those children of non-Finnish origin were excluded from the study. Additionally, for Cameriere’s method, 13 DPTs were excluded from the sample since 7 teeth to be examined had closed apices. While comparing dental ages of Finnish and Turkish children, 5-year-old Finnish children were excluded. To assess the population-specific formula, 54 DPTs of 29 girls and 25 boys were analysed by Cameriere’s method.

**Table 1:** Distribution of the participants according to sex.

Group	Boys	Girls	Total
Finnish	256 (53.1%)	226 (46.9%)	482 (100.0%)
Turkish	197 (46.6%)	226 (53.4%)	423 (100.0%)
Total	443 (49.5%)	452 (50.5%)	895 (100.0%)

### Turkish study sample

Turkish sample was randomly chosen from the dental archives of Faculty of Dentistry at Hacettepe University. The sample consisted of 423 DPTs of Turkish children, born between 2004 and 2014. Children’s age range was 6–15 years. Since Cameriere’s method cannot be used if all apices of the teeth are closed, one DPT was excluded from the sample.

### Study protocol

The same researcher (AD) assessed all radiographs. In Finland, a computer aided drafting software (Romexis, Planmeca, Helsinki, Finland) was used and images were analysed in a dark room designed for X-ray evaluation on two 24-inch monitors. In Türkiye, DPTs were assessed by Image J (IMAGE J 1.53, Wayna Rasband, NIH, USA) drafting software.

In both study populations, chronological ages were calculated by subtracting the exposure day from the day of birth of each individual by the software. Analyses of DPTs were done by AD who was blind to the patients’ age and gender.

For the Cameriere’s method, lower left teeth were assessed. The number of teeth with closed apices was calculated (N_0_), the teeth with open apices were assessed separately as follows. For teeth with one root, distances (mm) between inner sides of the apex were measured (Ai, i:1, 2, 3, 4, 5). For teeth with two roots, distances between the apices were measured and the sum of the distances were noted (Ai, i: 6, 7). The distances (Ai, i; 1, 2, 3, 4, 5, 6, 7) were divided by the tooth length (Li, i:1, 2, 3, 4, 5, 6, 7) to normalise the measurements as suggested by Cameriere et al. [1].

For Demirjian’s method, left mandibular teeth were assessed [3]. Developmental stages (A-H) were assigned to seven teeth according to their level of maturation. Scores of teeth were determined according to self-weighted scores table by using the developmental stages. The maturity scores were converted into dental ages for boys and girls, separately [30].

For intra-examiner reproducibility, 49 radiographs were re-evaluated by AD 4 weeks after the baseline assessment. A senior researcher (VA) and a senior radiologist (AS) were blinded to the study protocol, and they had been previously calibrated. Ten radiographs were evaluated by VA for inter-examiner reproducibility.

### Statistical analysis

To evaluate the intra- and inter-observer agreements, kappa statistic was used for Demirjian’s method. To assess Cameriere’s method, intraclass correlation coefficient (ICC) was used. Intra- and inter-observer agreements were obtained for each tooth.

Means and standard deviations (SD) were used to describe the distribution of the age estimated by Cameriere’s and Demirjian’s methods. Comparison of the mean difference between the estimated and chronological age was evaluated and the difference between two means was analysed by using paired *t*-test at 95% confidence interval (CI). The difference between the groups was considered statistically significant at the level *p* < 0.05. Pearson correlation coefficients were also used to estimate the correlation between the chronological and estimated age. Cross tabulation was used to illustrate the convenience of the methods chosen. Population specific regression coefficient estimates, and 95% CIs were calculated for Cameriere’s regression formula. Comparison of the two populations was conducted by mean differences of dental and chronological ages using paired-t test at 95% CI. All analyses were executed, and figures were drawn out using R software (version 4.0.2, a language and environment for statistical computing, R Foundation for Statistical Computing, Vienna, Austria, URL http://www.R-project.org).

## Results

The study was conducted on a total of 482 and 423 DPTs of the Finnish and Turkish children, respectively. For the assessment of population-specific-model, 54 DPTs of Finnish children were also included. For Demirjian’s method, intra-observer agreement was between 0.79 and 0.96, and inter-observer agreement was between 0.41 and 0.84. For Cameriere’s method, the intra-observer agreement was between 0.57 and 0.99, and inter-observer agreement was between 0.65 and 0.99.

### Finnish sample

For the Finnish sample, mean chronological ages were 9.85 ± 2.05 and 9.5 ± 2.01 for boys and girls, respectively. Mean estimated age of boys with Demirjian’s method were 10.32 ± 2.35 and 9.72 ± 2.13 for girls (Table 2). For Cameriere’s method, the same figures were 9.33 ± 1.72 and 8.96 ± 1.72 for boys and girls, respectively (Table 3). By both methods, children’s dental age differed significantly with respect to their chronological age (*p* < 0.0001). Demirjian’s method resulted in overestimation in all age groups except for 8-year-old girls. Dental age, however, was found to be underestimated with Cameriere’s method in all age groups but 6-year-old girls and boys. Although, Demirjian’s method led to better estimation than Cameriere’s method for girls, Cameriere’s method resulted in better prediction for boys than Demirjian’s method (Tables 2 and 3).

**Table 2:** Mean differences between dental age by Demirjian’s method and chronological age according to the age groups and sex distribution of the Finnish sample.

Chronological age					Dental age (Demirjian)		Difference between chronological and dental age					ME	
Age group	Sex	*n*	Mean	SD	Mean	SD	Mean	SD	Median	Min	Max	Mean	SD
6–6.99	Boys	12	6.67	0.28	7.63	0.37	0.96	0.41	0.98	0.22	1.66	0.96	0.41
	Girls	16	6.60	0.30	7.44	0.37	0.83	0.26	0.96	0.33	1.07	0.83	0.26
	Total	28	6.63	0.29	7.52	0.38	0.89	0.33	0.98	0.22	1.66	0.89	0.33
7–7.99	Boys	37	7.59	0.24	8.09	0.34	0.49	0.40	0.43	−0.41	1.30	0.53	0.35
	Girls	45	7.59	0.26	7.99	0.64	0.40	0.64	0.24	−0.71	2.21	0.51	0.55
	Total	82	7.59	0.25	8.03	0.52	0.44	0.54	0.35	−0.71	2.21	0.52	0.46
8–8.99	Boys	55	8.44	0.32	8.80	0.85	0.35	0.81	0.29	−0.90	3.35	0.63	0.62
	Girls	48	8.49	0.28	8.39	0.65	−0.10	0.62	−0.23	−1.40	1.43	0.51	0.37
	Total	103	8.47	0.30	8.61	0.79	0.14	0.76	−0.02	−1.40	3.35	0.57	0.52
9–9.99	Boys	45	9.50	0.29	9.73	0.96	0.24	0.99	0.14	−1.43	3.40	0.81	0.61
	Girls	36	9.50	0.27	9.71	0.70	0.21	0.71	0.13	−1.45	1.59	0.53	0.50
	Total	81	9.50	0.28	9.72	0.85	0.23	0.87	0.14	−1.45	3.40	0.69	0.58
10–10.99	Boys	35	10.49	0.32	10.94	0.97	0.45	0.94	0.56	−1.26	3.17	0.81	0.63
	Girls	32	10.54	0.30	10.78	1.10	0.24	1.01	0.37	−2.14	2.63	0.81	0.64
	Total	67	10.51	0.31	10.86	1.03	0.35	0.97	0.40	−2.14	3.17	0.81	0.63
11–11.99	Boys	33	11.51	0.31	11.68	1.26	0.17	1.20	0.09	−2.51	3.48	0.86	0.85
	Girls	22	11.50	0.29	11.59	1.49	0.08	1.35	−0.06	−3.04	2.32	1.04	0.84
	Total	55	11.51	0.30	11.65	1.34	0.14	1.25	0.04	−3.04	3.48	0.93	0.84
12–12.99	Boys	15	12.53	0.33	13.61	1.87	1.08	1.68	0.78	−1.44	3.29	1.57	1.20
	Girls	11	12.42	0.27	12.79	0.94	0.37	0.84	0.59	−1.08	1.52	0.76	0.46
	Total	26	12.48	0.30	13.26	1.58	0.78	1.41	0.66	−1.44	3.29	1.23	1.03
13–13.99	Boys	10	13.29	0.24	14.20	1.45	0.91	1.36	0.80	−0.64	2.52	1.28	0.97
	Girls	9	13.54	0.23	13.47	2.02	−0.08	1.96	0.75	−4.18	2.12	1.50	1.15
	Total	19	13.41	0.26	13.85	1.73	0.44	1.70	0.75	−4.18	2.52	1.39	1.04
14–14.99	Boys	14	14.41	0.31	15.39	0.96	0.97	1.01	1.27	−1.11	1.98	1.27	0.56
	Girls	7	14.54	0.29	14.84	0.86	0.31	0.76	0.06	−0.41	1.48	0.52	0.60
	Total	21	14.45	0.30	15.20	0.94	0.75	0.97	1.19	−1.11	1.98	1.02	0.66
Total	Boys	256	9.85	2.05	10.32	2.35	0.47	0.99	0.39	−2.51	3.48	0.83	0.72
	Girls	226	9.5	2.01	9.72	2.13	0.22	0.89	0.16	−4.18	2.63	0.68	0.62
	Total	482	9.69	2.03	10.04	2.27	0.35	0.96	0.29	−4.18	3.48	0.76	0.67

ME: mean prediction error; SD: standard deviation.

**Table 3:** Mean differences between dental age by Cameriere’s method and chronological age according to the age groups and sex distribution of the Finnish sample.

Chronological age					Dental age (Cameriere)		Difference between chronological and dental age					ME	
Age group	Sex	*n*	Mean	SD	Mean	SD	Mean	SD	Median	Min	Max	Mean	SD
6–6.99	Boys	12	6.67	0.28	7.16	0.27	0.49	0.33	0.55	0.06	1.20	0.49	0.33
	Girls	16	6.60	0.30	7.01	0.25	0.41	0.30	0.54	−0.15	0.89	0.45	0.24
	Total	28	6.63	0.29	7.08	0.26	0.45	0.31	0.54	−0.15	1.20	0.47	0.28
7–7.99	Boys	37	7.59	0.24	7.53	0.44	−0.06	0.46	−0.10	−0.79	1.25	0.36	0.28
	Girls	45	7.59	0.26	7.46	0.70	−0.13	0.71	−0.31	−1.11	2.05	0.57	0.43
	Total	82	7.59	0.25	7.49	0.59	−0.10	0.61	−0.17	−1.11	2.05	0.48	0.39
8–8.99	Boys	55	8.44	0.32	8.25	0.83	−0.20	0.80	−0.33	−1.37	1.98	0.68	0.46
	Girls	48	8.49	0.28	7.91	0.78	−0.59	0.77	−0.86	−2.17	1.54	0.81	0.52
	Total	103	8.47	0.30	8.09	0.82	−0.38	0.81	−0.57	−2.17	1.98	0.74	0.49
9–9.99	Boys	45	9.50	0.29	9.23	0.95	−0.27	0.94	−0.19	−2.38	1.92	0.79	0.57
	Girls	36	9.50	0.27	9.27	0.71	−0.22	0.73	−0.15	−2.38	1.08	0.58	0.48
	Total	81	9.50	0.28	9.25	0.84	−0.25	0.85	−0.16	−2.38	1.92	0.70	0.54
10–10.99	Boys	35	10.49	0.32	10.16	0.68	−0.32	0.69	−0.27	−1.60	1.82	0.55	0.51
	Girls	32	10.54	0.30	9.98	0.83	−0.55	0.77	−0.48	−2.65	1.52	0.72	0.61
	Total	67	10.51	0.31	10.08	0.76	−0.43	0.73	−0.33	−2.65	1.82	0.63	0.56
11–11.99	Boys	33	11.51	0.31	10.64	0.86	−0.88	0.81	−1.11	−2.30	1.76	1.11	0.44
	Girls	22	11.50	0.29	10.59	1.22	−0.91	1.09	−1.00	−3.68	0.67	1.16	0.81
	Total	55	11.51	0.30	11.62	1.01	−0.89	0.92	−1.04	−3.68	1.76	1.13	0.61
12–12.99	Boys	14	12.50	0.32	11.83	1.38	−0.67	1.23	−1.15	−2.10	1.20	1.21	0.65
	Girls	11	12.42	0.27	11.39	0.79	−1.04	0.68	−0.82	−2.14	−0.16	1.04	0.68
	Total	25	12.47	0.30	11.64	1.16	−0.83	1.03	−1.03	−2.14	1.20	1.13	0.66
13–13.99	Boys	9	13.26	0.24	12.35	1.07	−0.92	1.01	−1.30	−2.36	0.34	1.06	0.84
	Girls	8	13.50	0.21	11.99	1.16	−1.51	1.14	−1.21	−3.05	0.34	1.52	1.12
	Total	17	13.38	0.25	12.18	1.09	−1.20	1.08	−1.30	−3.05	0.34	1.28	0.98
14–14.99	Boys	6	14.44	0.31	12.85	0.72	−1.59	0.74	−1.67	−2.49	−0.58	1.59	0.74
	Girls	5	14.50	0.29	12.54	0.86	−1.96	0.56	−1.98	−0.79	−1.28	1.96	0.56
	Total	11	14.47	0.31	12.71	0.76	−1.76	0.66	−1.98	−2.79	−0.58	1.76	0.66
Total	Boys	246	9.68	1.89	9.33	1.72	−0.35	0.87	−0.32	−2.49	1.98	0.75	0.57
	Girls	223	9.44	1.94	8.96	1.72	−0.48	0.89	−0.46	−3.68	2.05	0.78	0.64
	Total	469	9.56	1.91	9.15	1.73	−0.41	0.88	−0.38	−3.68	2.05	0.77	0.60

ME: Mean prediction error; SD: standard deviation.

With Demirjian’s method, the percentages of boys and girls who had more than 2 years difference between the chronological and dental age were 6.8% and 4.7%, respectively. For Cameriere’s European formula, they were 3.5% and 6.1% for boys and girls, respectively. For both methods, more variations were observed in older individuals. Since the differences between chronological and dental age were significant, a specific model for Finnish population was developed for using the Cameriere’s method:

*Age* = 9*.*989 + 0*.*206*g* − 0*.*490*x*_5_ + 0*.*602*N*_0_ − 0*.*986*s* − 0*.*203*s* · *N*_0_

By using this population specific model and another sample, more than 2 years difference between the chronological age (CA) and dental age (DA) was noted for 6.9% and 8% of boys and girls, respectively. However, with the European formula, 2 years differences were found in 10.3% and 12.0% of boys and girls, respectively (Table 4).

**Table 4:** Mean differences of chronological and dental ages by Cameriere’s European formula and population-specific formula (Test sample).

Differences (years)	Boys	Girls	Total
Cameriere (European formula)			
<1	62.1 (18)	44.0 (11)	53.7 (29)
1–2	27.6 (8)	44.0 (11)	35.2 (19)
>2	10.3 (3)	12.0 (3)	11.1 (6)
Total	100.0 (29)	100.0 (25)	100.0 (54)
Cameriere (Population-specific formula)			
<1	62.1 (18)	68.0 (17)	64.8 (35)
1–2	31.0 (9)	24.0 (6)	27.8 (15)
>2	6.9 (2)	8.0 (2)	7.4 (4)
Total	100.0 (29)	100.0 (25)	100.0 (54)

### Turkish sample

For the Turkish sample, the mean estimated ages of boys and girls with Demirjian’s method were 10.59 ± 2.55 years and 10.75 ± 2.56 years, respectively (Table 5). The Demirjian’s method showed overestimation except for 14–14.99 years girls’ group. Mean differences between dental and chronological age for boys and girls were 0.64 ± 0.94 years and 0.55 ± 0.94 years, respectively. Using the Demirjian’s method, the biggest difference was found in 10–10.99 boys’ group (0.92 ± 0.6). In 9–9.99 girls’ group, the smallest difference was noted as 0.11 ± 1.10 years.

**Table 5:** Mean differences between dental age (by Demirjian’s method) and chronological age according to the age groups and sex distribution of the Turkish sample.

Chronological age					Dental age		Difference between chronological and dental age					ME	
Age group	Sex	*n*	Mean	SD	Mean	SD	Mean	SD	Median	Min	Max	Mean	SD
6–6.99	Boys	21	6.47	0.26	7.37	0.38	0.89	0.42	1.05	−0.02	1.54	0.90	0.42
	Girls	25	6.42	0.31	7.29	0.41	0.87	0.52	0.93	−0.54	1.72	0.95	0.36
	Total	46	6.44	0.29	7.33	0.39	0.88	0.48	0.94	−0.54	1.72	0.92	0.38
7–7.99	Boys	25	7.37	0.26	7.97	0.32	0.60	0.38	0.66	−0.14	1.39	0.62	0.35
	Girls	26	7.42	0.27	7.85	0.40	0.43	0.34	0.33	−0.07	1.38	0.44	0.34
	Total	51	7.39	0.26	7.91	0.37	0.52	0.37	0.39	−0.14	1.39	0.53	0.35
8–8.99	Boys	29	8.37	0.21	8.88	0.87	0.50	0.81	0.22	−0.40	2.62	0.65	0.69
	Girls	22	8.46	0.34	8.82	0.84	0.36	0.81	0.32	−0.73	2.07	0.66	0.58
	Total	51	8.41	0.27	8.85	0.85	0.44	0.80	0.32	−0.73	2.62	0.65	0.64
9–9.99	Boys	23	9.29	0.24	9.45	0.86	0.16	0.94	0.15	−1.37	2.16	0.71	0.62
	Girls	28	9.45	0.25	9.56	1.09	0.11	1.10	0.20	−1.87	2.24	0.94	0.55
	Total	51	9.38	0.26	9.51	0.98	0.13	1.02	0.15	−1.87	2.24	0.83	0.59
10–10.99	Boys	25	10.54	0.27	11.46	0.63	0.92	0.60	0.98	−0.78	1.96	0.98	0.48
	Girls	38	10.55	0.27	11.19	1.21	0.65	1.15	0.82	−2.29	3.48	1.07	0.76
	Total	63	10.55	0.27	11.30	1.02	0.75	0.97	0.88	−2.29	3.48	1.03	0.66
11–11.99	Boys	30	11.48	0.26	12.19	1.46	0.71	1.43	0.37	−1.13	4.18	1.11	1.14
	Girls	26	11.41	0.23	11.75	0.88	0.34	0.93	0.34	−1.53	2.89	0.76	0.63
	Total	56	11.45	0.25	11.98	1.23	0.54	1.23	0.35	−1.53	4.18	0.95	0.95
12–12.99	Boys	18	12.45	0.29	13.09	1.07	0.64	1.15	0.28	−0.96	3.42	0.94	0.91
	Girls	27	12.40	0.28	13.45	1.11	1.05	1.14	1.42	−1.14	2.52	1.33	0.79
	Total	45	12.42	0.28	13.30	1.09	0.89	1.15	1.10	−1.14	3.42	1.17	0.85
13–13.99	Boys	16	13.51	0.29	14.19	1.19	0.67	1.15	0.78	−1.58	2.58	1.12	0.69
	Girls	25	13.44	0.32	14.25	0.79	0.81	0.81	0.87	−2.25	1.57	1.01	0.53
	Total	41	13.47	0.31	14.23	0.95	0.76	0.94	0.87	−2.25	2.58	1.05	0.59
14–14.99	Boys	9	14.46	0.28	15.08	0.74	0.62	0.99	1.20	−0.58	1.69	0.99	0.57
	Girls	9	14.62	0.26	14.49	0.22	−0.13	0.35	−0.16	−0.77	0.37	0.30	0.20
	Total	18	14.54	0.28	14.78	0.61	0.25	0.82	−0.15	−0.77	1.69	0.64	0.54
Total	Boys	197	9.95	2.38	10.59	2.55	0.64	0.94	0.65	−1.58	4.18	0.87	0.72
	Girls	226	10.19	2.38	10.75	2.56	0.55	0.94	0.53	−2.29	3.48	0.88	0.64
	Total	423	10.08	2.38	10.67	2.55	0.59	0.94	0.59	−2.29	4.18	0.88	0.68

ME: Mean prediction error; SD: standard deviation.

Estimated ages of Turkish boys and girls with the Cameriere’s method were 9.72 ± 2.02 years and 9.94 ± 2.14 years, respectively. The Cameriere’s method showed underestimation in all groups except for 6–6.99 years and 7–7.99 years age groups (Table 6). The mean underestimations were 0.24 ± 0.99 years and 0.23 ± 0.91 years for boys and girls, respectively. The smallest difference between dental and chronological age was found in 8–8.99 years age group, being 0.03 ± 0.81 years for boys and 0.03 ± 1.02 years for girls. The biggest differences were found in 14–14.99 years age group (1.21 ± 0.75 years for boys, 1.44 ± 0.51 years for girls).

**Table 6:** Mean differences between dental age (by Cameriere’s method) and chronological age according to the age groups and sex distribution of the Turkish sample.

Chronological age					Dental age		Difference between chronological and dental age					ME	
Age group	Sex	*n*	Mean	SD	Mean	SD	Mean	SD	Median	Min	Max	Mean	SD
6–6.99	Boys	22	6.45	0.27	7.16	0.45	0.70	0.54	0.77	−0.81	1.56	0.82	0.31
	Girls	25	6.42	0.31	6.98	0.36	0.56	0.47	0.66	−0.38	1.72	0.63	0.37
	Total	47	6.44	0.29	7.06	0.41	0.63	0.50	0.71	−0.81	1.72	0.72	0.35
7–7.99	Boys	25	7.37	0.26	7.49	0.19	0.12	0.31	0.20	−0.52	0.74	0.29	0.16
	Girls	26	7.42	0.27	7.50	0.50	0.09	0.42	0.07	−0.72	1.23	0.29	0.31
	Total	51	7.39	0.26	7.50	0.38	0.10	0.37	0.12	−0.72	1.23	0.29	0.24
8–8.99	Boys	29	8.37	0.21	8.34	0.88	−0.03	0.81	−0.30	−1.52	1.70	0.66	0.46
	Girls	22	8.46	0.34	8.43	1.11	−0.03	1.02	−0.33	−1.80	2.14	0.83	0.56
	Total	51	8.41	0.27	8.38	0.97	−0.03	0.90	−0.30	−1.80	2.14	0.73	0.51
9–9.99	Boys	23	9.29	0.24	9.14	0.93	−0.14	1.02	−0.11	−1.85	1.52	0.85	0.55
	Girls	28	9.45	0.25	9.33	1.06	−0.12	1.05	0.15	−2.47	1.74	0.88	0.56
	Total	51	9.38	0.26	9.25	1.00	−0.13	1.02	0.00	−2.47	1.74	0.87	0.55
10–10.11	Boys	25	10.54	0.27	10.58	0.50	0.04	0.56	0.09	−1.59	1.37	0.39	0.40
	Girls	38	10.55	0.27	10.25	0.88	−0.30	0.81	−0.20	−2.49	1.79	0.57	0.64
	Total	63	10.55	0.27	10.38	0.77	−0.17	0.74	−0.09	−2.49	1.79	0.50	0.56
11–11.99	Boys	30	11.48	0.26	11.04	0.96	−0.44	0.93	−0.68	−1.52	2.06	0.86	0.55
	Girls	26	11.41	0.23	10.66	0.58	−0.75	0.63	−0.71	−1.94	1.10	0.84	0.49
	Total	56	11.45	0.25	10.86	0.82	−0.58	0.81	−0.71	−1.94	2.06	0.85	0.52
12–12.99	Boys	18	12.45	0.29	11.33	1.20	−1.12	1.27	−1.29	−4.30	1.33	1.36	0.99
	Girls	27	12.40	0.28	11.99	1.14	−0.40	1.14	−0.02	−2.40	1.33	0.95	0.73
	Total	45	12.42	0.28	11.73	1.20	−0.69	1.23	−0.66	−4.30	1.33	1.12	0.86
13–13.99	Boys	16	13.51	0.29	12.42	1.01	−1.10	0.98	−0.85	−2.59	0.49	1.16	0.90
	Girls	25	13.44	0.32	12.97	0.67	−0.47	0.72	−0.42	−2.59	0.35	0.64	0.57
	Total	41	13.47	0.31	12.76	0.85	−0.71	0.87	−0.53	−2.59	0.49	0.84	0.75
14–14.99	Boys	9	14.46	0.28	13.24	0.49	−1.21	0.75	−0.84	−2.13	0.36	1.21	0.75
	Girls	8	14.57	0.24	13.13	0.41	−1.44	0.51	−1.39	−2.39	−0.88	1.44	0.51
	Total	17	14.51	0.26	13.19	0.44	−1.32	0.64	−1.37	−2.39	−0.36	1.32	0.64
Total	Boys	197	9.95	2.38	9.72	2.02	−0.24	0.99	−0.2	−4.3	2.06	0.78	0.65
	Girls	225	10.17	2.36	9.94	2.14	−0.23	0.91	−0.2	−2.59	2.14	0.72	0.59
	Total	422	10.07	2.37	9.83	2.08	−0.24	0.94	−0.2	−4.3	2.14	0.75	0.62

ME: Mean prediction error; SD: standard deviation.

### Comparison of populations

When the estimated ages obtained by the Demirjian’s method were compared with chronological ages, it was seen that the estimated dental ages were higher in both sexes and all age groups with the exception of 8–8.99 years and 13–13.99 years age groups of Finnish girls and 14–14.99 years age group of Turkish girls. When the total data of Turkish and Finnish children were compared, the differences between the estimated age and chronological age obtained by the Demirjian’s method were found to be greater in Turkish children. The difference between the girls was greater than that of the boys (Table 7).

**Table 7:** Comparison of mean differences between dental age obtained by Demirjian’s method and chronological age according to the age groups and sex of Finnish and Turkish children.

Mean difference of DA-CA					
Age group (year)	Finland	Türkiye	*t*-value	*P*-value	95% CI
**Boys**					
6–6.99	0.965	0.894	0.472	0.641	−0.240, 0.382
7–7.99	0.494	0.603	−1.073	0.288	−0.312, 0.094
8–8.99	0.353	0.502	−0.802	0.426	−0.521, 0.223
9–9.99	0.236	0.162	0.303	0.763	−0.418, 0.567
10–10.99	0.450	0.920	−2.375	0.021^*^	−0.867, −0.074
11–11.99	0.170	0.713	−1.622	0.110	−1.214, 0.128
12–12.99	1.076	0.638	0.855	0.401	−0.619, 1.495
13–13.99	0.914	0.673	0.465	0.648	−0.851, 1.332
14–14.99	0.971	0.623	0.818	0.425	−0.549, 1.247
Total	0.469	0.638	−1.853	0.065	−0.349, 0.010
**Girls**					
6–6.99	0.834	0.871	−0.296	0.769	−0.287, 0.214
7–7.99	0.396	0.435	−0.330	0.742	−0.271, 0.194
8–8.99	−0.102	0.362	−2.385	0.023^*^	−0.860,−0.068
9–9.99	0.211	0.107	0.436	0.665	−0.377, 0.584
10–10.99	0.238	0.645	−1.572	0.121	−0.924, 0.110
11–11.99	0.085	0.338	−0.741	0.464	−0.945, 0.439
12–12.99	0.369	1.051	−2.034	0.053	−1.373, 0.008
13–13.99	−0.077	0.811	−1.316	0.221	−2.412, 0.638
14–14.99	0.308	−0.131	1.418	0.194	−0.274, 1.150
Total	0.216	0.552	−3.883	0.0001^*^	−0.506, −0.166

* Statistical significance (*p* < 0.05).

CI: confidence interval; DA: dental age; CA: chronological age.

As regard to the estimation of ages with the Cameriere’s method compared with the chronological ages, it was seen that estimated ages were lower in both sexes and all age groups, except for the 6-year-old boys and girls in both populations, and for 7-year-old boys in Turkish group. When the total data of the Turkish and Finnish children were compared, the differences between the estimated and chronological age obtained by the Cameriere’s method were found to be greater in Finnish children. The difference between the girls were greater than that of the boys in both populations (Table 8).

**Table 8:** Comparison of mean differences between dental age obtained by Cameriere’s method and chronological age according to the age groups and sex of the Finnish and Turkish children.

Mean difference of DA-CA					
Age group (year)	Finland	Türkiye	*t*-value	*P*-value	95% CI
**Boys**					
6–6.99	0.494	0.705	−1.413	0.168	−0.516, 0.094
7–7.99	−0.058	0.122	−1.838	0.071	−0.376, 0.016
8–8.99	−0.196	−0.033	−0.882	0.382	−0.534, 0.208
9–9.99	−0.270	−0.145	−0.491	0.626	−0.638, 0.388
10–10.99	−0.323	0.040	−2.241	0.029*	−0.687, −0.040
11–11.99	−0.877	−0.439	−1.984	0.052	−0.880, 0.004
12–12.99	−0.672	−1.124	1.014	0.319	−0.460, 1.363
13–13.99	−0.917	−1.098	0.435	0.669	−0.698, 1.059
14–14.99	−1.588	−1.215	−0.949	0.363	−1.238, 0.492
Total	−0.352	−0.237	−1.287	0.199	−0.291, 0.061
**Girls**					
6–6.99	0.411	0.561	−1.241	0.222	−0.392, 0.094
7–7.99	−0.134	0.087	−1.651	0.103	−0.488, 0.046
8–8.99	−0.587	−0.034	−2.262	0.030*	−1.052, −0.055
9–9.99	−0.222	−0.119	−0.445	0.658	−0.572, 0.365
10–10.99	−0.554	−0.300	−1.346	0.183	−0.630, 0.123
11–11.99	−0.914	−0.751	−0.621	0.539	−0.699, 0.373
12–12.99	−1.035	−0.402	−2.104	0.044*	−1.247, −0.019
13–13.99	−1.510	−0.468	−2.441	0.038*	−2.008, −0.074
14–14.99	−1.958	−1.439	−1.691	0.130	−1.228, 0.190
Total	−0.479	−0.235	−2.871	0.004*	−0.411, −0.077

* Statistical significance (*p* < 0.05).

CI: confidence interval; DA: dental age; CA: chronological age.

When Turkish and Finnish children were compared, the differences between dental age obtained by the Demirjian’s method and chronological age were found to be significantly different at 10 years of age for boys and 8 years of age for girls (Table 7). The differences between estimated ages obtained by the Cameriere’s method and chronological ages were significantly different in 10-year-old boys and 8-, 12- and 13-year-old girls (*p* < 0.05) (Table 8).

## Discussion

The demand for age estimation of living individuals by courts for criminal cases, adoptions, asylum-seekers, refugees, and immigrants is increasing [19]. There is also requirement for postmortem investigations [31]. After the Southeast Asian Tsunami on 26 December 2004, age estimation became most important. Among 5,395 victims of the disaster, 55 were Finnish individuals below 18 years of age from different parts of Finland [31]. There are limited number of Finnish studies on dental age estimation [18, 20, 32, 33]. However, there are two recent articles for Turkish children [21, 29]. Again, after disasters like earthquakes in Asia Minor, dental age estimation is required. Hence, the present study aimed to contribute to literature in this respect. The present study is the first to report on the Cameriere’s method in a Finnish population. The Demirjian’s method was found to be more suitable for the Northern Finnish boys while the Cameriere’s method was more suitable for girls in the Northern Finland. The Finnish population-specific model developed here, is more accurate when the Cameriere’s method is employed. However, the formula was evaluated in a small sample. In order to verify the results, bigger sample sizes should be achieved. Underestimation of the age must be taken into consideration when European or population-specific formula is used for the Cameriere’s method. When populations were compared, Turkish children showed advanced tooth maturation. The differences were significant in girls.

Different tooth development rates have been observed in different ethnic origins [34]. Studies have shown that children living in different regions of a country might also have differences in their dental development [35]. It has been reported earlier that children living in the southern and northeastern parts of Finland had showed different tooth maturation patterns [36]. In a Turkish study, it was stated that the eastern, north-eastern, and northern Turkish children had more advanced dental development than that of children living in the western Turkey [37]. Additionally, the present generations showed different maturation patterns when compared to the previous ones [20].

Several types of age estimation methods that involve skeletal or dental status of individuals are available [31]. Using levels of developmental tooth calcification to determine chronological age has several advantages [38]. Tooth development is controlled by genes and is less affected by endocrinological diseases or environmental factors [19, 20]. These methods are based on radiography and as such reproducible and easy to use [32]. Counting erupted deciduous teeth can also be used for age estimation but it is limited to toddlers. On the other hand, counting erupted permanent teeth for older children and adolescents may be affected by extensive caries in primary dentition and early extractions [33]. The present study examined the calcification stages of teeth in radiographs by two different methods.

In literature, there are studies comparing dental development of geographically and culturally different populations or different groups within the same population [36, 39–41]. There is no published study which compared dental development of Finnish and Turkish populations. The present study compared the accuracy of dental age estimation methods in Turkish and Finnish children as well as the differences between two nationalities. The study is also relevant when immigration to the west, that is, from Türkiye to Finland is increasing. Data and research from countries with large emigration are important if later age estimation is needed in the country of immigration. It has been stated that the use of proportional data provided convenience for using panoramic radiographs from different centres [42].

Although the Cameriere’s method has not been assessed in Finland, it has been used in many countries such as Italy, Colombia, Malaysia, Serbia [1, 24, 43–45], and even in Turkey [29]. The method has shown a tendency of underestimation of dental age [22], which was reported for Turkish [43] and Serbian children [24], and Bosnia-Herzegovinian boys [23]. Cameriere et al. [46] compared their method with the Demirjian’s method in a study with children of ages between 5 and 15 years form Italia, Spain, and Croatia. They reported that their method slightly underestimated chronological age, while the Demirjian’s method presented an overestimation. Those findings are in line with the present study. The authors concluded that the better age estimation was achievable with their method. In a meta-analysis, however, it was stated that there was no difference between boys and girls [47]. The study was conducted by Cameriere et al. [46] who showed better age estimation for boys than girls (not statistically significant). Similarly, in the present study, better age estimation was obtained with the Cameriere’s method for Finnish boys than girls within the same age range. For all that, in Turkish children, the Cameriere’s method showed better estimation than the Demirjian’s method for both sexes. Another study, conducted in a German population, comparing the Cameriere’s and Demirjian’s methods reported that Demirjian’s method was advantageous for both sexes [25]. These different results in different populations may suggest that age determination must be population-specific [25]. Present study results also indicated that the Demirjian’s and Cameriere’s methods lead to better estimation in younger ages. The findings were found to be similar to those in previous studies [46, 48]. High accuracy in younger individuals may be attributable to multiple different developmental stages at the same time in younger children.

Reliability of the Cameriere’s European Formula was evaluated in Turkish children by Gulsahi et al. [43]. The results showed 0.35 year of underestimation of chronological age. The difference was found to be 0.47 and 0.24 years for boys and girls, respectively. In our study, using the same method in a similar population from the same city, the mean differences between the estimated and chronological ages were 0.24 ± 0.99 years for boys and 0.23 ± 0.92 years for girls. These results showed that Cameriere method was an advantageous method in terms of reproducibility. However, dental age was underestimated by the European formula in a study conducted in Northern Germany (0.32 ± 0.96 years for boys and 0.56 ± 1.04 years for girls). In the study, the North Germany formula was adapted, and a new sample was evaluated with the adapted formula. New formula underestimated age of the boys and girls only 0.04 ± 80 years and 0.08 ± 0.83 years, respectively [49]. Similarly, when the population-specific model was used in our study, the difference between chronological age and estimated age decreased both in girls and in boys. These results again indicated the benefit using population-specific methods.

A study with the Finnish children using the Demirjian’s method showed overestimation (0.29 years for males; 0.43 years for females) [50] which was parallel to the current results. Another study analysing DARLInG method which was based on the Demirjian’s method including Northern Finnish participants and examining secular trend showed 0.34 ± 0.87 years of overestimation and 3.15 ± 1.58 years of underestimation in their present and early groups, respectively [20]. Similarly, in the present study dental age was overestimated by 0.35 ± 0.96 years with the Demirjian’s method. That shows the consistency of the Demirjian’s method, although studies were conducted with modifications of the Demirjian’s method. Age determination of South Asian Tsunami disaster victims has been performed in a study comparing the skeletal and dental age estimation methods. The smallest deviation between the chronological age and the estimated age was reported in the Demirjian’s method and methods based on eruption of teeth. However, the biggest limitation of the study has been reported as the small number of samples [31].

In the current study, estimated ages with the Demirjian’s method were greater than chronological ages in all age groups. For all that, the differences between the estimated and chronological ages were greater in the Turkish than Finnish children for both sexes. On the other hand, in the Finnish children, underestimation was more pronounced than that of the Turkish children. These results suggested that the Turkish children had more advanced dental development between the ages of 6 and 15 compared to the Finnish children.

When these two populations were compared, differences between the chronological and estimated ages obtained by the Demirjian’s method were significantly different in 10-year-old boys and 8-year-old girls. As for the Cameriere’s method, the differences between the chronological and dental age were significant in 10-year-old boys and 8-,12-, and 13-year-old girls. Significant differences were found in boys and girls two years apart between two populations. Similarly, onset of puberty occurs 2 years apart in girls and boys. Bundak et al. [51] have reported the mean age of menarche for Turkish girls as 12.2 ± 0.9 years in their study. In a Finnish study, the age of menarche was reported as 13.16 ± 0.02 years [52]. The significant differences between dental maturation and chronological age in the 12–13 age group in the Turkish and Finnish girls are similar when the ages of menarche were compared, and, therefore, may explain the results.

As for the strengths, the present study was conducted in two fairly large study populations. This has allowed comparison of different methods as well as dental age of two ethnically different child populations. Another advantage was that one trained and calibrated dentist with good intra- and inter-examiner agreement analysed all DPTs. However, one examiner in the study could also be stated as a study limitation.

The present study offered scientific evidence on the estimation of dental age of both sexes in two ethnically different populations by utilising different radiographic methods. The findings suggested that compared to a group of Finnish children, Turkish children had more advanced dental development between the ages of 6 and 15 years. Differences between chronological and estimated ages obtained by the Demirjian’s and Cameriere’s methods were significantly different in 10-year-old boys and 8-year-old girls, and also in 12-, and 13-year-old girls by Cameriere’s method. The results indicated the need for population specific models in estimation of children’s dental age, whenever they are available. In forensic medicine, specific methods should be used in different populations and genders determined by the literature. A model created here for the Finnish child population seems to have better accuracy than the European one, and can be beneficial in clinical use.

## Geolocation information

Finland and Türkiye.
